# “How difficult it is to change dietary behaviour” experience of older people with sarcopenic obesity: a qualitative study

**DOI:** 10.1186/s12877-024-05157-0

**Published:** 2024-07-01

**Authors:** Yue-Heng Yin, Justina Yat Wa Liu, Maritta Välimäki

**Affiliations:** 1 School of Nursing, Nanjing Medical University, 101 Long Mian Avenue, Nanjing, 211166 China; 2 School of Nursing, The Hong Kong Polytechnic University, Hong Kong SAR, China; 3 Faculty of Medicine, Department of Public Health, University of Helsinki, Helsinki, Finland; 4 Turku, Finland

**Keywords:** Dietary behavioural change, Barriers, Facilitators, Qualitative study

## Abstract

**Background:**

Dietary intervention is an important method to manage sarcopenic obesity, but the implementation in real world is difficult to achieve an ideal condition. This study aimed to the experiences of older people with sarcopenic obesity during the implementation of dietary behavioural change (DBC) intervention.

**Methods:**

This study is a semi-structured individual interview embedded within a pilot randomized controlled trial on community-dwelling older people with sarcopenic obesity. Purposive sampling was applied to invite 21 participants who had received a 15-week DBC intervention. The interviews were audio-recorded and transcribed verbatim. Content analysis was performed to analyze the data.

**Results:**

The themes for facilitators included: (a) Attach importance to self’s health; (b) Family’s support; (c) Concern self’s body shape; (d) Instructor’s support; (e) Regular food diary taken. The themes for barriers included: (a) Difficulties of taking food diary; (b) Difficulties of calculating the food amount; (c) Yield to offspring’s appetite; (d) Misjudging self’s or family’s appetite.

**Conclusion:**

Support from family members and instructor, caring about self’s health and body image facilitated the intervention implementation. The complication of food amount estimation and diary record, personal sacrifice for next generations, and previous living experience were barriers for implementing the intervention. Overall, the older people with sarcopenic obesity can accept the design of DBC intervention program and have great willing to join.

## Background

Sarcopenic obesity is a physical condition in the ageing process where fat accumulation is simultaneously accompanied by muscle loss [1]. Research has reported sarcopenic obesity can lead to adverse health outcomes, significantly increasing the risk of developing fatigue, physical disability, poor quality of life, institutionalisation, morbidity, and mortality [2]. The prevalence of sarcopenic obesity in China ranged from 3.2 to 20.4% in women and 13.8–27.0% in men [3].

Dietary intervention by emphasizing the high-quality protein intake and caloric restriction is found to be an important non-pharmacological intervention on managing sarcopenic obesity [4]. To date, only two studies have been conducted using solely nutritional intervention and have yielded inconsistent findings [5, 6]. The effective doses of protein intake and caloric restriction for sarcopenic obese older people are still unclear. According to the systematic review we conducted, calorie intake of a 12% reduction and protein intake of 1.2–1.5 g/kg body weight/day may be an effective dietary intervention method for preventing sarcopenic obesity [2]. In addition, the sustaining behavioural change for the participants in current research about dietary intervention was difficult [7]. Methods like behavioural change techniques should be incorporated into the study design to improve participants’ adherence to the dietary intervention, because the longer the study, the more the challenges of maintaining participants’ compliance increase [8]. Behaviour change techniques (BCTs) used in an intervention usually mean the elements to modify or divert causal processes that direct behaviour, which can be observed and replicated [9]. The BCTs may contain the characteristics like self-monitoring, problem-solving, coping plan, persuasive argument, review behaviour goals, behavioural rehearsal, etc. Effective BCTs have been commonly used to change dietary habits, promote physical activity, and smoking cessation [10–12]. Therefore, dietary interventions combined with BCTs can be a good solution for managing sarcopenic obesity on older people.

The implementation of dietary behavioural change (DBC) intervention among older people with sarcopenic obesity in the real world has never been investigated before. It is necessary to explore the experiences of older adults during the dietary behaviour change intervention process, which is extremely important for the success of the interventional project. The qualitative research can not only help understand the implementation of the intervention, but also help explore the experience of participants who undertaken the dietary intervention trial, which could help us understand more deeply about how the intervention works, how the participants feel, and then contribute to the modification of intervention design and implementation process. Considering this, a qualitative study was designed to embedded within a pilot trial of DBC intervention among olde people with sarcopenic obesity.

## Methods

### Design

This qualitative study was designed as semi-structured individual interviews embedded within a pilot randomized controlled trial (RCT). This pilot RCT has been described elsewhere [13], in brief, it was a two-armed parallel trial aimed to evaluate the effects of DBC intervention on managing sarcopenic obesity. There were 30 participants in the experimental and control group, respectively. The participants in the experimental group received a 15-week dietary intervention combined with behaviour change techniques guided by the Health Action Process Approach model [14], and they were required to record food diary during the intervention.

Participants from the experimental group participated in this qualitative study after the completion of intervention. The 32-item checklist Consolidated Criteria for Reporting Qualitative Research (COREQ) was referred to report the qualitative study [15], and this study was registered in (ClinicalTrial.gov: NCT04690985).

### Study setting and recruitment

The participants were recruited by purposive sampling from the community healthcare centre in Nanjing, China between June 2020 to February 2021. Nanjing is the capital city of Jiangsu Province with an excellent economic level in China. The study was promoted by displaying the posters in the centre. Potential participants were identified by the community nurses, and community physician ascertained whether the participant’s physical health condition was suitable for the study. If an older adult was eligible for the study, we would provide the information sheet and ask for consent.

### Participants

For the participants enrolled in the interventional study, the community physician and nurses helped the research assistants to screen and diagnose potential participants. The inclusion criteria were community-dwelling older people who: (a) were aged 60 years or above; (b) met the condition of sarcopenic obesity according to the Asian Working Group for Sarcopenia (AWGS) 2019 [1] and China’s guidelines for obesity [16], which included: (i) handgrip strength lower than 28 kg for men and lower than 18 kg for women, or physical performance reflected by 5-time chair stand test ≥ 12 s; (ii) BMI ≥ 28%, or waist circumference ≥ 85 cm in men and ≥ 80 cm in women; (c) were able to communicate, read, and write in Chinese without significant hearing and vision problems to ensure they understand our instructions.

The exclusion criteria were older people who: (a) were already adhering to special diet restrictions, including diabetes-specific diet, ketogenic diet, which may affect the digestion of protein or energy metabolism; (b) had been diagnosed with alcohol addiction, which was an important factor for obesity;

I had already been taken part in other clinical studies; (d) had any medical implant devices such as pacemakers; I took medications that may influence digestion or metabolism; (f) were suffering from metabolic disorders (e.g., renal diseases, diabetes), or any other diseases/conditions which may affect food intake and digestion.

The participants from the experimental group were purposively recruited into the qualitative review based on their levels of adherence to food diary taking to balance the proportion of people with good and poor compliance. The final number of participants were determined by the data saturation.

### Data collection

Qualitative data was collected within two weeks after completing the intervention among participants from the experimental group based on their levels of adherence to food diary taking. The interviews were conducted in a private meeting room in the community centre to avoid disturbance from other people, and the participants are already familiar with the environment. The interviewees were informed about the purpose and procedures of the interview, then the questions in Table 1 were asked. At the end of the interview, an open-ended question was asked to obtain further information which might be relevant: “Is there anything else you would like to tell me?”. The whole interview process was audio-taped, and field notes were taken to record the non-verbal reactions of the interviewees.

**Table 1:** Open-ended questions asked in the semi-structured interview

Items	Questions
Q1	How do you feel about the whole dietary behaviour change process?
Q2	To what extent do you think the intervention is helpful for you?
Q3	What factors do you think facilitated your change in dietary behaviour?
Q4	Then what factors do you think hindered your change in dietary behaviour?
Q5	Was your motivation increased after the first two face-to-face meetings?
Q6	Was the dietary plan helpful for you to turn the motivation into action?
Q7	Did your family support your dietary behaviour change? If not, why?
Q8	Now the study has finished, will you consider keeping this dietary behaviour? If not, could you please tell me the reasons?
Q9	In your opinion, what are the strengths and weaknesses of the intervention? Do you have any suggestions?

### Data analysis

Content analysis was employed inductively to analyse the qualitative data, which is a flexible method for analysing text data and has been widely used in qualitative studies [17]. The field notes were also analysed. After each interview, the interviewer transcribed the voice record into text immediately. A research assistant helped with the transcription. Two researchers checked the accuracy of the transcripts together. NVivo 12 software was used to manage the data to help identify common codes from the transcripts. Two researchers worked independently on the coding and identifying of the codes. The research team then discussed the identified codes and came to an agreement on them, grouping them into categories with the support of verbatim data. Then, the categories were condensed and/or reorganised when deemed necessary. The research team examined the categories for consistency, reached a consensus about the meaning and structure, and devised a list of finalised categories. A set of themes with supporting verbatim data were finally generated to identify the perceptions of participants.

### Ethical considerations

The ethical approval was obtained from the Human Subjects Ethics Review Committee of the Hong Kong Polytechnic University (HSEARS20191007001). Written informed consent was obtained from each participant after being given the study’s introduction. We reassured each participant that they could withdraw from the study at any time without penalties. The principle of protecting research subjects was observed in accordance with the Helsinki Declaration. Data confidentiality was maintained in accordance with the privacy ordinance.

### Rigor and reflexivity

The rigor of this study was guaranteed according to the four criteria proposed by Guba and Lincoln [18]: credibility, transferability, dependability, and confirmability. (i) Credibility: The interview outline had received two-round of revision based on experts’ comments. A pilot interview was conducted ahead of time to find potential problems. The interviewer is equipped with professional ability to conduct the interviews. The peer debriefing was completed by double-checking the transcripts, themes, and categories by two researchers separately and then comparing the similarities and differences. (ii) Transferability: The participants were purposive sampled from the community who can represented the common older people with unhealthy dietary behaviour. The data saturation was guaranteed during the interview to obtain the maximum of information [19]. A clear and distinct description of the settings, characteristics of participants, data collection, analysis process, and appropriate quotations were given in this manuscript. (iii) Dependability: The data including the original transcripts and coding trees were rechecked with participants to ensure that the meaning expressed accurately represented their perceptions. The whole coding process was reviewed by two experienced researchers. Iv) Confirmability: A detailed record of the research process including data collection and analysis were kept allowing for transparency and accountability.

The researchers had critically examined their own beliefs, values, and biases that may impact the research, and they always held a critical stance and honesty to ensure the interpretation was based on the data [20]. The researchers wrote memos during data analysis to record the thoughts and reflections on the data to help addressing potential biases or preconceived notions.

## Results

### Participants

A total of 21 participants (mean age: 68.19 ± 6.30 years old) were involved in the semi-structured individual interviews. The adherence of the interviewees for food diary taking were diverse as good (7/21), moderate (1/21), and bad (13/21). The adherence to recording the food diary was rated as ‘good’, ‘moderate’, or ‘bad’, according to the average reports on frequency: ‘6–7 days/week’, ‘3–5 days/week’, and ‘0–2 days/week’, respectively. Details of the characteristics of the participants are shown in Table 2.

**Table 2:** Demographics of interviewees (*n* = 21)

Demographics	Count (%)
**Gender**	
Female	14 (66.7%)
Male	7 (33.3)
**Marital status**	
Married	19 (90.5%)
Single/Divorced/Widow	2 (9.5%)
**Education level**	
Primary school	4 (19.0%)
Secondary school	8 (38.1%)
High school	8 (38.1)
Diploma/Bachelor degree or above	1 (4.8%)
**Religious belief**	
Christ	2 (9.5%)
Buddhist	1 (4.8%)
None	18 (85.7%)
**Household monthly income (CNY)**	
< 3,000	4 (19.0%)
3,000–5,999	7 (33.3%)
6,000–10,000	6 (28.6%)
> 10,000	4 (19.0%)
**Vegetarian**	
No	19 (90.5%)
Yes	2 (9.5%)

### Findings

Overall, the interviewees felt the DBC intervention was helpful to promote their health, they were motivated to change dietary behaviour, and the arrangements of intervention sessions were acceptable. The participants felt the DBC program was meaningful in helping them distinguish the correctness of online nutrition information in terms of knowing better about the food type and daily recommended food amount. All interviewees felt a desire to change their dietary behaviour after joining the accelerating courses.

Two themes were identified regarding the participant’s feelings of joining the DBC intervention program: (1) facilitators for implementing the DBC intervention, and (2) barriers in participating in the DBC intervention. The themes and sub-themes are listed in the Table 3.

**Table 3:** Themes and sub-themes generated from the content analysis

Themes	Sub-themes	Code units
Facilitators of dietary behavioural change	a. Attach importance to self’s health.	• Worried that nobody could take care of them if they fell ill. • Know the importance of diet and health.
	b. Family’s support.	• Received encouragement from their partners or children
	c. Concern self’s body shape.	• Care about the beauty of wearing clothes. • Concerned about the fat accumulation in the abdomen and legs.
	d. Instructor’s support.	• The instructor changed their mind about eating, helped solve problems, gave encouragement.
	e. Regular food diary taken.	• Self-monitoring daily route
Barriers of dietary behavioural change	a. Difficulties of taking food diary.	• Easy forget, too much housework, low education level
	b. Difficulties of calculating the food amount.	• Cannot estimate the food amount accurately • Difficulty of food amount estimation caused by the dining style
	c. Yield to offspring’s appetite.	• Prioritize the taste preferences of children when preparing the food • Give children the best quality of food rather than themselves
	d. Misjudging self’s or family’s appetite.	• Cook excessive amount of food • Be hesitant to discard leftover food

### Theme 1: facilitators of dietary behavioural change

#### Sub-theme a: attach importance to self’s health

The most mentioned facilitators for participating in the DBC intervention were that interviewees cared about themselves’ health and did not want to add burden to their children. Parts of the interviewees live with their spouses, and their children are busy without having time to look after them, so they are worried that nobody could take care of them if they fell ill. In addition, after attending the first two sessions, they all have the sense that sarcopenic obesity may lead to bad health outcomes, and diet has strong relations to sarcopenic obesity. Therefore, they were very willing to learn the nutritional knowledge and adherence to what they were taught in the sessions.

*Our children are not around. If my husband and I get sick, no one can take care of us. We have to take care of ourselves, so we care about daily diet quality. (P1)*

*This year my husband passed away. I learnt a lesson from his bad living habits. Especially after taking your courses, I am more aware of the importance of a healthy diet. (P9)*

*My legs are painful when I walk, and I feel a little burdensome. It was inconvenient on many occasions when I was fat. For example, it was difficult and uncomfortable to tie shoes and bend over. So, I am willing to change previous lifestyle. (P16)*

*Early this year, I had cerebral infarction. Before that, I thought my body was perfect, but then I doubted it. I started to control my weight and pay attention to my food intake. (P20)*

#### Sub-theme b: family’s support

The support from the family members was another crucial facilitator for the interviewees participating in the DBC intervention. The interviewees presented that they received encouragement from their partners or children, and some family members even are motivated to pay attention to their dietary behaviours.

*My family support me to do so. My daughter even said, mom, you seem to be thinner this year than last year. I also need to pay attention to my weight. (P3)*

*My family support me to control my diet. My husband was even afraid that I would not insist on it. He continued to remind me. (P21)*

#### Sub-theme c: concern self’s body shape

Interviewees’ attention to their image also motivates them to change their dietary behaviours, which both male and female interviewees mentioned. They were more concerned about the fat accumulation in the abdomen and legs. Some female interviewees were concerned about the effects of fat body shape on the beauty of wearing clothes.

*When I was young, I had been at 60 kg for at least 20 or 30 years. With the advantage of age, my belly has become looser than before. There are so many beautiful clothes that I can’t wear in summer. (P1, female)*

*Because my body is fat without too much muscle, it is puffiness. From the deep of my heart, I want to get rid of the annoying fat. (P5, male)*

#### Sub-theme d: instructor’s support

The behavioural change techniques used by the diet instructor was helpful for participants to change their dietary behaviour. The participants mentioned that the support from the diet instructor was essential for them to insist on the dietary behaviour change. First, the diet instructor changed their mind about eating. Before joining the program, they were unaware of sarcopenic obesity and did not pay attention to muscle function. Instead, they paid more attention to controlling the salt or sugar in daily meals to avoid hypertension and diabetes. After joining the program, they realised the risk of sarcopenic obesity and the critical influence of muscle mass on physical function, motivating them to change their dietary behaviour. Second, the diet instructor helped them solve problems encountered during the intervention process. The encouragement from the diet instructor gave them strong support to overcome the barriers (mentioned above in *Theme 2*). For example, they learnt to distinguish food types such as carbohydrates (e.g., sweet potato) under the guidance of the diet instructor, which gave them the confidence to continue the dietary plan. They also gained confidence and achievements from the decrease in body weight.

*What you said is inspiring for me, and your courses are pretty good (P3).*

*I couldn’t distinguish between staple food and tables before. Now that I know what food is the staple food, I start to control the intake amount. My weight has also lost five or six catties. I am lucky to meet you, and it gives me a lot of strength (P9).*

#### Sub-theme e: regular food diary taken

Interestingly, some interviewees liked the food diary taken because it could help them to better plan daily meals, which could facilitate them to insist on implementing the diet plan.

*Recording the food makes my dietary pattern much clearer. For example, if I feel I have not eaten the meat for a long time, but I couldn’t recall how long it is, I just check the food diary, and I’ll know when I have eaten it, then I will plan when to buy meat in the following days. (P1)*

*Taking food diary let me feel good. After all, I don’t have anything to do since I retired, and I don’t have too many activities every day. Taking food diary let me have stuffs to do every day. (P5)*

### Theme 2: barriers of dietary behavioural change

#### Sub-theme a: difficulties of taking food diary

Most of the interviewees presented that they have barriers in keeping the food diary, the reasons of which were: being busy looking after grandchildren, easy to forget, forgetting how to write the food’s name in characters, and feeling no need to take food diary due to a regular daily eating pattern.

*To be honest, we are not very well educated, and sometimes the characters of the foods name cannot be recalled and written. Besides, it’s (taking food diary) just like taking medicine, although the doctor told me to take medication every day, I would forget it occasionally. (P5)*

*Taking care of grandchildren has delayed a lot of things. I am too busy to sit down, so I miss recording the food very often. (P6)*

*In fact, it is not too troublesome. I eat similar foods regularly every day, and there are no big differences. Even if I take notes of what I eat, it is almost the same every day. I can remember what I eat, so I feel it is not necessary to write it down. (P7)*

#### Sub-theme b: difficulties of calculating the food amount

Quite a few interviewees reported it was difficult for them to estimate the food amount even though each of them had been given a food scale and taught how to use it. The Chinese dining style made it is challenging to estimate each one’s eating amount of food, and they were unlikely to adopt a split meal system.

*It’s not easy to control the amount of food. I feel that I can eat as much as I want. I eat when I’m hungry, and I stop eating when I am full. There are no specific standards for me. (P8)*

*A packaged food, for example, a cake, I can put it on the scale and weigh it. But for a bowl of cooked rice, I can’t weigh it before eating and then weigh it after eating. It seems that I have no such habit. It is troublesome, and the food amount can only be estimated. (P5)*

#### Sub-theme c: yield to offspring’s appetite

Most of the interviewees lived together with their children to help look after the grandchildren. They tended to put their children’s and grandchildren’s taste in the priority instead of themselves. Only one interviewee chose to cook and eat alone because he could not accommodate himself to the children’s appetite.

*The obstacle is that our family eats together, which means I eat with my daughter, son-in-law, and grandchildren. So I seldom consider planning my own diet well. (P3)*

*My husband and I like to eat fish, but our children do not like it. Considering the children, we don’t buy fish very often. (P7)*

*We always consider the children’s appetite when we prepare the food instead of ourselves. (P20)*

#### Sub-theme d: misjudging self’s or family’s appetite

Not willing to waste leftovers is another barrier to dietary behaviour change. Most interviewees chose to eat overnight leftovers, especially for the meat, because they all experienced hard times when they were young. The poor quality of life in the past still affects their current life habits, making them think wasting food is terrible. However, this phenomenon of leftovers was improved because they learnt to control the food amount when cooking.

*We were poor when we were young, so I feel it is too wasteful to pour the leftovers. I always eat it all. Now I gradually start to change my bad behaviour after joining your program, and I cook less food. (P3)*

*We never waste leftovers. My grandma died at the age of 96, and she ate the leftovers. My mother died at the age of 92, and she also ate the leftovers. I believe that everyone’s physique is different. (P7)*

## Discussion

To the best of our knowledge, this is the first study to explore the barriers and facilitators of implementing evidence-based dietary behavioural change interventions on people with sarcopenic obesity, the population of whom had a dilemma of dietary intake, which means they need to intake rich proteins to increase muscle but meanwhile they need to limit the caloric intake because of the obese condition. Previous studies on dietary intervention showed contradictory results. This study fills the gap by exploring the deep reasons of dietary behavioural change among sarcopenic obese older people in terms of elucidating the facilitators and barriers during implementation.

According to the qualitative interview, the behaviour change techniques may play an important role in facilitating participants’ acceptability of the DBC intervention. The continuous support by the interventionist during the intervention process was reported as a crucial factor for behaviour change. Previous studies aimed for lifestyle modification usually focused on providing knowledge, materials, and professional education, which may be insufficient to make any behavioural changes [21]. Instead, providing alternative strategies to deal with obstacles in actual practice may be more effective to change the behaviour, such as promoting participants’ awareness of risk behaviour or facilitating participants’ self-monitoring [14].

Specific Chinese contextual culture may bring some barriers to the participant’s acceptability of the DBC intervention. For example, Chinese older people are usually expected to take care of their grandchildren. Due to time constraints, they may be reluctant to participate in health promotion programmes [22]. According to our qualitative interview, the acceptability of the DBC intervention was mainly affected by internal (e.g., previous eating habits, forgetfulness, laziness) and external factors (e.g., economic level, the complexity of food recording, specific Chinese culture). Among these factors, Chinese culture, family mode, and dining style played important roles in participants’ acceptance of the DBC intervention. We will discuss more about the cultural influences on dietary behaviour change in the following paragraphs.

First, the participants’ previous personal experiences brought great barriers to changing their dietary behaviour. The participants, who experienced poor lives, lacked the awareness of control the amount when preparing the food, which leading to excess cooked food. They were reluctant to waste food including leftovers, and usually eat up all the food, leading to excessive food intake. This exposure in participants’ early lives greatly impacts eating habits in subsequent adulthood [23, 24].

Second, the traditional Chinese family mode is another critical barrier in changing participants’ dietary behaviour. According to our qualitative review, the common barrier for preventing dietary behaviour change was that the participants needed to take care of grandchildren. They had no enough time to plan their own diet, and they put the children’s taste prior to themselves. We also found grandparents always sacrifice their own needs in the choice of food types. Other researchers also reported this kind of phenomenon, young children have the priority to receive food, and their food quality is always the best, which was consistent with our interview results [25].

Third, Chinese families usually eat together. This sharing dining culture makes older people seldom split meals. Most Chinese families eat in a grouped dining system because sharing food is regarded as a close relationship and atmosphere within a Chinese family [25], which is different from Western’s separate dining style. Therefore, it would be difficult to let the participants eat separately with their families, and it caused difficulties for participants to control the amount of food intake. Because the cooking methods of Chinese dishes are complicated, it is not easy to calculate the exact amount of food intake if without splitting meals. In addition, the effect of cooking and food preparation skills may also affect the individual’s compliance especially for those who were not good at cooking.

Interestingly, food diary taken is found to be both facilitator and barrier of dietary behavioural change. For some older people, food diary taken enable them get rid of a mundane daily routine and help them clarify daily food intake, which facilitate them to insist on the dietary behaviour. Previous study has also reported self-regulatory behavior change technique is an essential factor for success diet intervention [26]. But the difficulties of food amount estimation hinder some older people’s passion for changing dietary behaviour. Previous studies have already tested various digital methods of food record such as image-based assessment applications, smart log supported by deep learning, wearable devices, etc. [27–29]. But valid methods with high accuracy are still under developing. Moreover, keeping the dietary records with the help from a nutrition professional may lead to more accurate information retrieval, a more convenient communication platform could be developed.

### Strengths and limitations

Strengths of this study are obvious. The diverse characteristics of the participants (e.g., age, gender, education level, economic level, etc.) and purposeful random sampling methods ensure the low selection bias of participants. The interventions were developed through evidence-based process and the design of the whole qualitative interview strictly followed the methodological guideline.

There are also limitations of this study. The interviewer and the participants were familiar with each other along with the advancement of the interventional project. The participants may hide their honest reflection of the intervention and cause participants bias (i.e., acquiescence bias or friendliness bias) [30]. We adopted the following strategies to reduce this effect: (a) open-ended questions were asked to avoid participants simply agreeing/disagreeing, and to provide the participants a range of options instead of simple ‘Yes’ or ‘No’; (b) the interviewer guided them to talk freely without concerns and to provide a truthful and honest answer; (c) the interviewer asked questions in different ways if the answers sounded not trustworthy.

### Implications for research and practice

The study had some implications in clinical practice. First, considering the adverse health outcomes aroused by sarcopenic obesity, the need of providing professional nutrition knowledge and interventions to older people in community-dwelling settings is pressing. Understanding the facilitators and barriers of implementing dietary intervention is beneficial for older people’s behaviour promotion. Second, community healthcare workers could play a role in implementing the DBC intervention, which has been proved to be a feasible and acceptable method to improve older people’s dietary quality. In our study, the participants produced a desire to change their dietary behaviour, overcome obstacles, and finally improve dietary quality. However, it was challenging for older people to change their behaviour by relying solely on self-control. Suppose community healthcare workers can play a supervisory role and use behaviour change techniques (e.g., workshops or telephone follow-up or online guidance), which may have a positive effect on helping older people change into healthier dietary behaviour.

This study can provide suggestions for future research. Interventions need to be better tailored to older Chinese people who could not balance their roles in taking care of grandchildren and planning their daily meals. Having family members’ support is essential [31]. In future studies, contextual factors need to be explored and considered, such as involving family members in the intervention process.

## Conclusion

Basically, the older people with sarcopenic obesity show great enthusiasms and willing to change their dietary behaviour to obtain a healthy body. They can accept the DBC intervention based on the positive feedback to the intervention. Support from family members and instructor is important to facilitate the older people changing their dietary behaviour. However, the compliance rate of recording a food diary and accurate estimation of food amount still have room for improvements. They also needed to be more egotistic when handling the role in the family.

## Data Availability

The data and materials are not publicly available as the participants did not consenting to share their data. Further details about the data and ethical conditions are available from the corresponding author on reasonable request.

## Abbreviations

AWGS: Asian Working Group for Sarcopenia
BCTs: Behaviour change techniques
COREQ: Consolidated Criteria for Reporting Qualitative Research
DBC: Dietary behavioural change
RCT: Randomized controlled trial
